# Adventures of psychiatry in a general hospital: a tale of C-L psychiatry origins and resistance in Brazil

**DOI:** 10.47626/1516-4446-2024-3854

**Published:** 2024-11-25

**Authors:** Juliano Victor Luna, João Alberto Carvalho

**Affiliations:** 1 Faculdade de Ciências Médicas, Universidade de Pernambuco, Recife, PE, Brazil; 2 Hospital das Clínicas, Universidade Federal de Pernambuco, Recife, PE, Brazil; 3 Centro de Ciências Médicas, Universidade Federal de Pernambuco, Recife, PE, Brazil

The first viable psychiatric ward in a general hospital was created in the United States in 1902, and by the 1920s, the movement toward integrating psychiatry and general hospitals had gained momentum.^1^ Around the world, consultation-liaison psychiatry (CLP) is present and recognized as a crucial part of psychiatric training, and its status as a psychiatric subspecialty has been consolidated.^1^ Several studies have shown that the availability of a CLP service improves the care provided to hospital patients, reduces length of hospital stay, and lowers hospitalization costs.^2^

Unfortunately, that is not the case in Brazil. In 2005, Brazil was the 125th country among 180 on the WHO’s list of mental health beds in general hospitals vs. total mental health beds. As of 2016, Brazil was 76th, but due to the closure of psychiatric hospitals rather than to an increase in mental health beds in general hospitals.^3^ The Brazilian mental health policy plays a central role in this problem. Although the Brazilian psychiatric reform movement was instrumental in improving several aspects of Brazilian mental health care, the idea of general hospital psychiatry is not privileged as a way forward in helping to advance deinstitutionalization.^4^

In 2020, the Brazilian Psychiatric Association published a directive emphasizing that “Mental health outpatient clinics and psychiatric units in general hospitals are indispensable for an effective network.” A psychiatric unit in a general hospital (PU-GH) should not be confused with simple “psychiatric beds” in general hospitals, unsupported by trained teams.^5^ Changes in Brazilian mental health policy are urgently needed, and general hospital psychiatry should play a vital role in this.

History is essential in cultivating memory and offering lessons. It was created under the auspices of Mnemosyne, the goddess of poetry and memory, and *mnémons* (remembrances, testimonies) were originally the pillars of historical narrative.^6^ Medicine and psychiatry are also historical constructs, reflecting human needs, mentalities, and conceptions across different cultures and times.^7^

Brazil’s first PU-GH was established in 1954 at the Hospital das Clínicas of the University of Bahia.^7^ Professor Nelson Pires, a member of the first generation of psychiatrists from the Social Psychiatry School of Recife and a direct disciple of Ulysses Pernambucano, coordinated its creation. In 1956, another member of this generation inaugurated the PU-GH at the Hospital das Clínicas of the Federal University of Pernambuco (UFPE).^7^

It is worth noting that CLP services have a rich history of successful experiences that have yet to be told. By presenting the history of UFPE’s PU-GH – one of the first Brazilian psychiatric units in a general hospital, which has been operating nonstop since 1956 despite arduous obstacles – we intend to add some insights to the debate surrounding the challenges to the effective progress of CLP in our country.

## A brief history of hospitals in Pernambuco

Public hospital care in Pernambuco was founded in the 16th century under the aegis of the Santas Casas de Misericórdia, hospitals administered by the Catholic Church. Until 1802, however, hospitals did not accept the mentally ill. Those had a different place in society: if harmless, they became the subject of mockery by children and street urchins; if agitated, they were taken to the public jail, held alongside murderers and thieves, or even executed as sorcerers or witches. If they belonged to a wealthy family, a “prison room” was built at the back of the house, where the troublesome patient was segregated until death.^8^

From 1802, the “alienated” were accepted into the medical care of the Santas Casas under the influence of Pinel’s work and subsequent paradigm shift. Not long after, however, the need to separate them from the rest of the patients became urgent. In 1861, Hospital Pedro II was inaugurated as the most important hospital in Pernambuco. The province’s president was keen to assure the population that the hospital, “based on the principles of science and the practices of cultured countries, is not to have them [patients] accompanied by the unfortunate lunatics with whom they lived in the old hospital.”^7^

A group of political leaders sought a different arrangement. After a hard-won fight involving satires in daily newspapers, a place in a distant rural area was designated to harbor an asylum, which was constructed – under much criticism – and inaugurated in 1883. This was the Tamarineira asylum, the birthplace of the Social Psychiatry School of Recife, led by Ulysses Pernambucano, and the first psychiatric teaching hospital in the North and Northeast regions of Brazil.^9^ It is now known as Hospital Ulysses Pernambucano, and the once-rural Tamarineira is one of the prime real estate areas in Recife. Most of the hospital grounds have since become a public park, and a movement is underway to shut down the hospital and turn it into a museum. But that is another story.

## Get back to where you (should have) once belonged: the PU-GH at Hospital Pedro II

Professor José Lucena, responsible for implementing the PU-GH at UFPE, was Ulysses Pernambucano’s formal successor. Lucena was appointed Chair of Psychiatry at UFPE in 1950. By then, Hospital Pedro II (later renamed Hospital das Clínicas) was the UFPE teaching hospital. Although the Tamarineira asylum did not cease to exist, Professor Lucena decided to transfer psychiatry teaching activities to Pedro II. To do so, he proposed and, not without difficulties, succeeded in creating a PU-GH there, which began operating in 1956.

In his own words: “It is natural that the proposal to create psychiatric units in general hospitals will meet strong resistance, but this is one of the measures through which we can give new impetus to modernizing psychiatric care in our environment.”^10^ Integration, however, was a more challenging goal to pursue.

The psychiatric clinic had 30 beds and was located behind the main hospital block, but not connected to it. At Pedro II, the lunatics had been housed alongside the consumptives’ ward and the kennel, which held the dogs used for experimental surgery. The importance of establishing a psychiatry residency and medical psychology teaching in the general hospital, which prevented the conversion of the psychiatric unit into a tiny sanatorium or “micro-asylum” embedded within the larger hospital structure, cannot be overstated.

Until his retirement as Chair of Psychiatry in 1978, Professor Lucena and his collaborators conducted several studies on PU-HGs. Their last study made a pioneering contribution regarding the outpatient dimension of CLP. They found that only 0.8% of patients seen by the UFPE Outpatient Psychiatric Clinic between 1970 and 1976 were referred from other hospital clinics, mostly internal medicine and neurology. The article concluded with a call for team integration and greater acceptance of psychiatric patients within the general hospital community.^11^ After 22 years, the space was being shared, but psychiatry was still kept firmly outside the hospital.

## Hospital das Clínicas is transferred to a new building: where shall we go?

In 1982, after a long construction period, UFPE’s Hospital das Clínicas was transferred to a brand-new building, tall and wide, with modernist architectural features. The accommodation process was not easy for Psychiatry. No predetermined space was allocated for the mentally ill, and many were against installing a psychiatric ward in this vertical structure, as it would pose a menace to the psychiatric patients’ integrity. Or was it the other way around?

If the move from Tamarineira to Pedro II was promising and a cause for celebration, the abrupt and untimely transfer of the HC to its new premises in the early 1980s was experienced by the healthcare team as genuinely traumatic, with genuine fear of reliving the Pedro II inauguration of 1861 more than a century later. Fortunately, after solid diplomatic efforts, an arrangement was made with the head of Internal Medicine, and the psychiatric ward was installed on the 7th floor, with 12 beds, side by side with the Internal Medicine ward. This arrangement persisted until 2007, when Internal Medicine expanded and moved to the 11th floor.

The psychiatric ward is still located on the 7th floor, and still houses the same 12 beds. The HC psychiatric service has developed other pioneering initiatives, such as an outpatient CLP clinic in the late 1980s. This unit, unprecedented in Brazil, was designed for patients with no prior psychiatric history who developed psychiatric symptoms acutely during a clinical or surgical hospitalization. To this day, the articulation between patient care, teaching, and research has been kept strong, and the Hospital remains a regional reference in Brazilian psychiatry.

## Perspectives and reflections

UFPE’s PU-GH has been functioning uninterruptedly since 1956. Despite being a training facility for undergraduate medical students and residents, it has experienced many obstacles to its continued operation, and probably would no longer exist if it were in a non-academic setting. It is one of Brazil’s few public healthcare services that offers ECT regularly, for instance.

We can arguably reason that the gap between psychiatry and other medical specialties has been closing in these 70 years. However, the stigma toward mentally ill patients and their presence in a general hospital has not been overcome. These difficulties are embedded in the Brazilian paradigm of mental health care, which resists incorporation of the general hospital as a legitimate setting for psychiatric care. Just as in Pernambuco, CLP has historically faced challenges in expanding throughout the Brazilian territory.^4^ Most CLP units are in academic facilities, such as the State University of Campinas (UNICAMP) and Federal University of São Paulo (UNIFESP) and Hospital Universitário Clementino Fraga Filho (HUCFF) at the Federal University of Rio de Janeiro (UFRJ), which are kept running for their importance as teaching centers and research output as much as for the patient care they provide.

When one tries to envision the integration of CLP and the Brazilian Mental Health Policy (RAPS), it still seems a far-fetched goal down a long, winding, two-way road of self-perpetuating resistance, which keeps PU-GH as islands and CLP as an adventure.

## Disclosure

The authors report no conflicts of interest.
